# The HMGB1-2 Ovarian Cancer Interactome. The Role of HMGB Proteins and Their Interacting Partners MIEN1 and NOP53 in Ovary Cancer and Drug-Response

**DOI:** 10.3390/cancers12092435

**Published:** 2020-08-27

**Authors:** María Cámara-Quílez, Aida Barreiro-Alonso, Ángel Vizoso-Vázquez, Esther Rodríguez-Belmonte, María Quindós-Varela, Mónica Lamas-Maceiras, María Esperanza Cerdán

**Affiliations:** 1 Department of Biology, EXPRELA Group, Centro de Investigacións Científicas Avanzadas (CICA), Departamento de Bioloxía, Facultade de Ciencias, INIBIC- Universidade da Coruña, Campus de A Coruña, 15071 A Coruña, Spain; maria.camara@udc.es (M.C.-Q.); aida.barreiro@udc.es (A.B.-A.); a.vizoso@udc.es (Á.V.-V.); belmonte@udc.es (E.R.-B.); 2 Department of Oncology, Translational Cancer Research Group, Instituto de Investigación Biomédica de A Coruña (INIBIC), Carretera del Pasaje s/n, 15006 A Coruña, Spain; Maria.Quindos.Varela@sergas.es

**Keywords:** ovarian cancer, interactome, chemotherapy

## Abstract

High mobility group box B (HMGB) proteins are overexpressed in different types of cancers such as epithelial ovarian cancers (EOC). We have determined the first interactome of HMGB1 and HMGB2 in epithelial ovarian cancer (the EOC-HMGB interactome). Libraries from the SKOV-3 cell line and a primary transitional cell carcinoma (TCC) ovarian tumor were tested by the Yeast Two Hybrid (Y2H) approach. The interactome reveals proteins that are related to cancer hallmarks and their expression is altered in EOC. Moreover, some of these proteins have been associated to survival and prognosis of patients. The interaction of MIEN1 and NOP53 with HMGB2 has been validated by co-immunoprecipitation in SKOV-3 and PEO1 cell lines. SKOV-3 cells were treated with different anti-tumoral drugs to evaluate changes in HMGB1, HMGB2, MIEN1 and NOP53 gene expression. Results show that combined treatment of paclitaxel and carboplatin induces a stronger down-regulation of these genes in comparison to individual treatments. Individual treatment with paclitaxel or olaparib up-regulates NOP53, which is expressed at lower levels in EOC than in non-cancerous cells. On the other hand, bevacizumab diminishes the expression of HMGB2 and NOP53. This study also shows that silencing of these genes affects cell-viability after drug exposure. HMGB1 silencing causes loss of response to paclitaxel, whereas silencing of HMGB2 slightly increases sensitivity to olaparib. Silencing of either HMGB1 or HMGB2 increases sensitivity to carboplatin. Lastly, a moderate loss of response to bevacizumab is observed when NOP53 is silenced.

## 1. Introduction

Ovarian cancer is nowadays the 7th most common cancer in women, with only a 30–40% average 5-year relative survival rate. Early diagnosis, when the tumor is still localized in the ovaries, is a clear advantage, since this rate then increases up to 92% [1]. More than 90% of malignant ovarian tumors are epithelial ovarian cancers (EOC) and the rest derive from stromal or germ cells. The histology of malignant EOC, or carcinomas, is heterogeneous [1] and they have been classified in five main histotypes: high-grade serous (HGSOC with an incidence of 70% among total EOC), low-grade serous (LGSOC; incidence <5%); endometrioid (ENOC; incidence of 10%), clear cell (CCOC; incidence of 10%), and mucinous (MOC; incidence 3%). This classification takes into account the resemblance to normal gynecological cell line; serous: resembling epithelium lining the Fallopian tubes; mucinous: resembling epithelium lining endocervix, and containing intra-cytoplasmic mucin; endometrioid: resembling epithelium of uterine corpus; clear cell: comprising clear cells and hobnail cells [1]. Each histotype has been associated to a particular set of somatic mutations. HGSOC type is associated to BRCA1/2 and TP53 mutations; LGSOC type, to BRAF and, KRAS mutations; MOC type, to KRAS mutation; ENOC type to PTEN, CTNNB1, ARID1A and PIK3CA mutations; and finally CCOC type, to ARID1A and PIK3CA mutations [1].

High mobility group box-B proteins (HMGB), non-histone components of chromatin, exert global regulatory functions on gene expression [2]. Their release from cancerous cells to the extracellular medium promotes tumor growth and metastasis, and their overexpression is associated to ovarian cancer, among others [3]. Increased HMGB1 expression has been associated to TLR4 expression and activated NF-κB signaling pathway [4,5]. This is accompanied by worse clinical outcomes in EOC patients, which suggests that signaling by endogenous ligands may contribute to an inflammatory microenvironment, which worsens the disease [4]. In the ovarian cancer cell line SKOV-3, HMGB1 silencing diminishes the expression of VEGF and CXCL12, revealing its involvement in angiogenesis [6].

HMGB1 has been repeatedly proposed as a diagnostic and prognostic biomarker for human ovarian cancer [7,8,9,10,11]. HMGB1 immunostaining in serous, mucinous, endometrioid, and clear-cell ovarian carcinomas [12] shows that HMGB1 expression is observed in different EOC histotypes. HMGB1 overexpression in early stages of the disease is also an advantage for its use as diagnostic biomarker [12]. HMGB1 and HMGB2 proteins have been also related to chemotherapy resistance in serous EOC [13]. High expression of HMGB1 has been related to cisplatin resistance [14] and downregulation of HMGB1 re-sensitizes ovarian cancer cells to carboplatin [15]. HMGB2 expression has been associated to resistance against oxaliplatin and cisplatin [16].

Protein interactomes associated to a particular disease are valuable tools to understand molecular mechanisms of pathogenesis and to re-define diagnostic panels of specific biomarkers [17]. The low rates of survival after first diagnosis, clearly show that clinical management of ovarian cancer patients needs of earlier diagnosis and more specific therapies [18,19]. Considering the relevance of HMGB proteins in EOC, deciphering the HMGB1/2 interactome in ovarian cancerous cells is an attractive tool to achieve this goal. We have followed a Yeast-Two-Hybrid (Y2H) approach using libraries derived from SKOV-3 cells, a widely used ovarian cancer cell line; and also libraries from cancer tissue from a patient diagnosed of primary transitional cell carcinoma (TCC) of the ovary, a relatively rare subtype of epithelial ovarian cancer, which represents approximately 2% of all ovarian tumors [20]. Common targets in both libraries could discover important interactions, which are independent of the histological subtypes and their specific mutations. Functional significance of the discovered targets in relation to ovarian cancer is discussed, with special focus on MIEN1 and NOP53. Expression changes of HMGB1, HMGB2, MIEN1 and NOP53 genes have been evaluated in response to drugs usually employed in ovarian cancer treatments: bevacizumab, olaparib, paclitaxel or carboplatin. The effects of HMGB1, HMGB2, MIEN1 and NOP53 silencing on cell sensitivity to these drugs are also reported.

## 2. Results

### 2.1. HMGB1 and HMGB2 Y2H-Interactomes in Epithelial Ovarian Cancer

Protein interactions were assayed by the yeast two-hybrid (Y2H) approach. The baits (HMGB1 or HMGB2) were cloned in fusion to the DNA-binding domain of the yeast transcriptional activator GAL4, while libraries of prey proteins were fused in frame to the GAL4 activation domain. Plasmids expressing bait and prey fusion proteins were used to transform two different haploid yeast strains able to form diploids. After mating, the transcriptional activator GAL4 is reconstructed in the diploids only if interaction between proteins exists, and therefore reporter genes under GAL4-activated promoters are expressed. This technique is highly sensitive and, in order to diminish the appearance of false positives, a screening using independent markers was used as explained in Materials and Methods. Two cDNA libraries were constructed using total RNA extracted from SKOV-3 cells or from tissue obtained from a tumor diagnosed as primary transitional cell carcinoma (TCC) of the ovary. TCC is grouped with high-grade serous carcinoma (HGSC) in the current World Health Organization classification and it is also associated with BRCA mutations [21]. Y2H assays and screening were carried out as described in Materials and Methods and clones, identified as positives using at least three different reporter genes, were sequenced. In the libraries derived from SKOV-3 cells and using the HMGB1 bait (Table 1), a positive clone showed homology to lncRNA MALAT1 and 5 to coding sequences (AKIP1, KRT7, ATF71P, UHRF2, WDR60); using the HMGB2 bait, 7 coding sequences were identified (BCCIP, COMMD1, NOP53, MIEN1, ROCK1, U2AF1, ZNF668).

In the library prepared from ovarian tumor tissue (Table 2), 5 clones with coding sequences were identified using the HMGB1 bait (C1QA, DAG1, RPL29, RSF1, TGM2), and 6 (COMMD1, MIEN1, PCBP1, TBC1D25, ZFR, ZNF428) were identified with the HMGB2 bait. Table 1 and Table 2 summarize the details of each detected interaction with HMGB1 or HMGB2, as well as a brief functional description of the proteins identified in these EOC-HMGB-interactomes.

As above reported, MALAT1, a lncRNA, previously related to ovarian cancer [22,23,24,25,26,27], and to epithelial to mesenchymal transition (EMT) [28], was detected in our results of EOC-HMGB1-interactome (Table 1). We verified by sequencing that the micropeptide derived from MALAT1 lncRNA, MTEVEMKLLHGVKNVFKRKLRERTTEPRINTNRRAMLLD, is in frame and fused to GAL4 in the recovered Y2H clone. Therefore, it is possible that the interaction of this micropeptide with HMGB1 might be responsible of the positive result obtained in the Y2H screening. In support of this explanation, translation of this micropeptide from lncRNA MALAT1 has been previously reported in several ribosome-profiling experiments using human colorectal cancer cells HCT116 [29] and human embryonic kidney HEK293 cells [30].

Two proteins that interact with HMGB2, COMMD1 and MIEN1, are identified in SKOV-3 and tumor tissue libraries, which cross-validate these results. The interactions of HMGB1 with RLP29 and ZNF428, and the interaction of HMGB2 with ZNF428 were already described in non-cancerous ovarian HOSEpiC cells from epithelial origin [31]. The interaction between HMGB1 and KRT7 was also detected in cells from healthy ovarian tissue [31].

**Figure 1:**
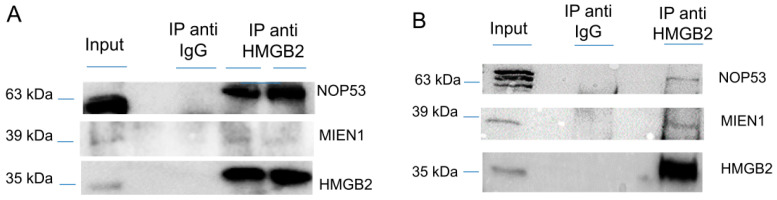
Co-immunoprecipitation of HMGB2 and MIEN1 or NOP53 (**A**) in SKOV-3 cells (**B**) in PEO1 cells. Proteins extracted from cells (Input) were immunoprecipitated with HMGB2 antibodies (IP anti HMMGB2) or IgG (IP anti IgG) and the Western blot were incubated with HMGB2, MIEN1 and NOP53 antibodies.

Since the Y2H interactome was obtained from SKOV-3 cells, not considered as a cell line representative of the more frequent HGSOC, although extensively used in EOC studies [32], and from tissue extracted from primary transitional cell carcinoma (TCC) of the ovary, which is a relatively unfrequently diagnosed serous EOC, we decided to validate HMGB2 interactions with MIEN1 and NOP53 by co-immunoprecipitation, an orthogonal method to the Y2H approach, and using two EOC cell lines, SKOV-3 and PEO1 representatives of ENOC/CCOC and HGSOC respectively [33]. Results (Figure 1 and Figure S1) corroborate the Y2H data. Apparently the co-immunoprecipitation of NOP53 with HMGB2 is much more efficient in SKOV-3 than in PEO1, perhaps due to the different characteristics of both cell lines, although we cannot attribute this observation to a specific cause.

**Figure 2:**
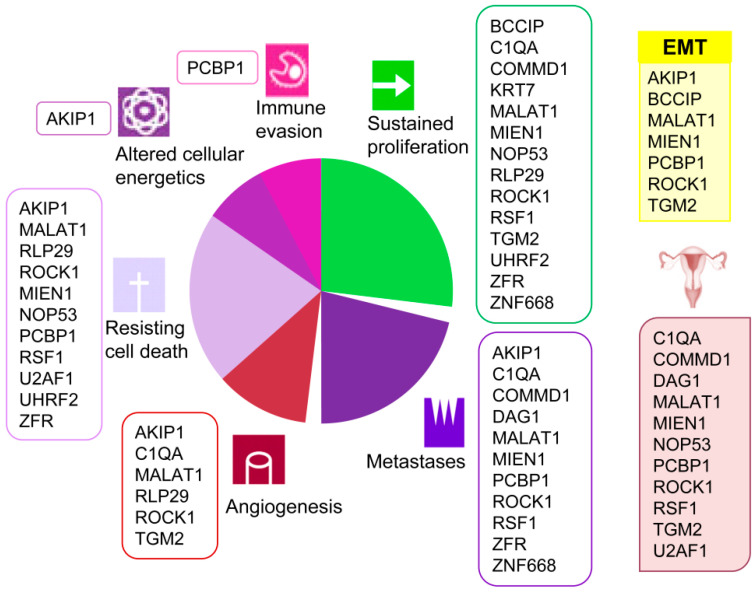
Associations of proteins detected in the Y2H EOC-HMGB-interactome to cancer hallmarks, EOC and EMT. Proteins previously related to cancer from ovarian origin are shown under the ovary pictogram; those involved in epithelial to mesenchymal transition under the yellow EMT box. References supporting the scheme are indicated for each protein as follows. AKIP1 [56,57,58,59,60], BCCIP [61,62], COMMD1 [63,64,65], C1QA [66], DAG1 [67], KRT7 [68], MALAT1 [24,69,70], MIEN1 [55,71,72], NOP53 [73,74,75], PCBP1 [76,77,78], RLP29 [79,80], ROCK1 [38,81,82,83,84,85,86], RSF1 [47,48,87,88], TGM2 [89,90], UHRF2 [91,92], U2AF1 [93], ZNF668 [94,95], ZRF [96,97,98].

A clear association of these proteins with several current cancer hallmarks such as sustained proliferation, metastasis, angiogenesis, resisting cell death, altered cellular energetics, and immune evasion is evidenced (Figure 2). Besides, and remarkably, several among these proteins have been previously associated to ovarian cancer (COMMD1 [34], NOP53 [35], MIEN1 [36,37], ROCK1 [38,39,40], PCBP1 [41], TGM2 [42], U2AF1 [43], C1QA [44], DAG1 [45] and RSF1 [46,47,48,49]). Furthermore, several detected proteins (BCCIP [50], ROCK1 [51], PCBP1 [52], AKIP1 [53], TGM2 [54], and MIEN1 [55]) are involved in the epithelial to mesenchymal transition (EMT), typical of epithelial cells in malignant differentiation processes. However, none of them had been cited for their interaction with HMGB proteins until now, which add value to the present work and reveals the importance of the HMGB interactome in EOC. We have reviewed in the literature the experimentally confirmed functions of the proteins detected in our EOC-HMGB-interactome study (Figure 2).

### 2.2. Analysis of the EOC-HMGB-Interactome According to Differential Expression and Clinical Outcome

**Figure 3:**
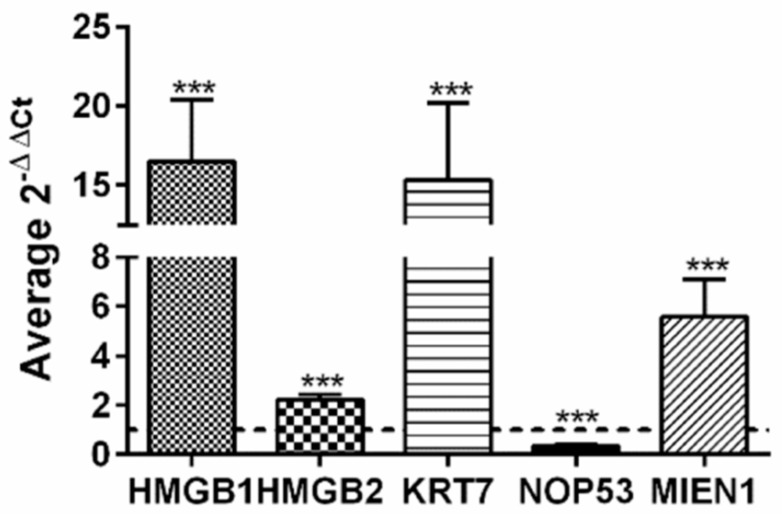
Relative expression of HMGB1, HMGB2, KRT7, MIEN1 and NOP53 genes in SKOV-3 cells versus non-cancerous human ovarian HOSEpiC cells. The dotted line indicates no variation, boxes upper the line show genes over-expressed in SKOV-3 cells, and those under the line are genes under-expressed in SKOV-3 cells. *** (*p* < 0.001).

Taking advantage of public available data, accessible though Expression Atlas [99] at European Bioinformatics Institute (https://www.ebi.ac.uk, accessed on 15-5-2020) we have compared gene expression levels of HMGB1, HMGB2 and all the genes found in our EOC-HMGB-interactome in ovary tissue from healthy individuals (39 samples from GTEx Project [100]) and public data extracted from Pan-Cancer Analysis of Whole Genomes (PCAWG) corresponding to 110 tumors of ovarian adenocarcinomas (Table 3). Data indicate that HMGB1, HMGB2, as well as most of their targets identified in our Y2H study are expressed at higher levels in ovarian adenocarcinoma than in normal ovarian tissue, following a pattern of co-regulation that is frequently found among genes encoding proteins that interact with each other [101]. The highest ratios of RNA changes in cancerous *versus* healthy ovarian samples correspond to KRT7 (ratio >1700), C1QA (ratio 12.5) and MIEN1 (ratio 6.3). Only two genes, NOP53 and MALAT1 are less expressed in cancerous than in healthy ovarian cells in this comparison. Selecting a subgroup of genes we have also directly observed in experiments carried in our laboratory that HMGB1, HMGB2, MIEN1 and KRT7 are expressed at higher levels in SKOV-3 cancerous cells than in HOSEpiC normal ovary cells (Figure 3); while, also in accordance with patient data (Table 3), NOP53 is expressed at lower levels in the cancerous cell line than in the healthy ovary cell line (Figure 3). Although it is generally accepted that the majority of HGSOC arise from an extra-ovarian site we wanted to discriminate non-cancerous ovarian cells from tumor cells. For this reason we preferred to use ovarian non-cancerous cells as a pair-matched control.

**Figure 4:**
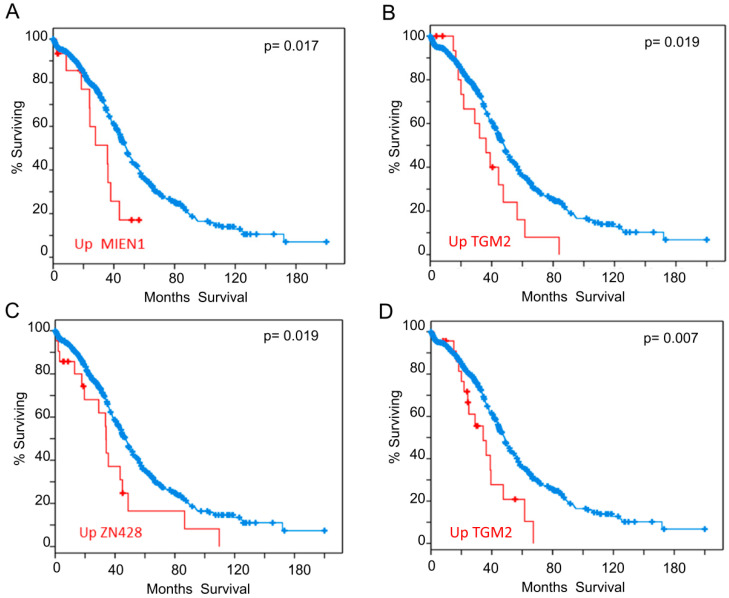
Overall Survival Kaplan-Meier Estimate. Significant differences found in the analysis carried out with the proteins from Table 1 and Table 2. (**A**) Influence of MIEN1 overexpression according to array data (**B**) Influence of TGM2 overexpression according to array data (**C**) Influence of ZN428 overexpression according to RNAseq data (**D**) Influence of TGM2 overexpression according to RNAseq data. Red line represents cases with alterations and blue line cases without alterations.

We also analyzed our interactome components (listed in Table 1 and Table 2) with the tools available in cBioportal (http://www.cbioportal.org/ accessed on 03-06-2020) [102,103] looking for correlations between expression levels and clinical outcome. To carry out this analysis, we selected samples from the study “Ovarian Serous Cystadenocarcinoma (TCGA)” with information of 606 samples from 594 patients (https://www.cbioportal.org/study/summary?id=ov_tcga. accessed on 03-06-2020). We found that patients who have higher expression of some of the genes identified in our study have lower survival expectation than the rest. Analysis of expression data based on microarray technology revealed that up-regulation of mRNA levels of MIEN1 (Figure 4A) or TGM2 (Figure 4B), negatively correlated to survival. Analysis of expression data based on RNAseq technology showed that up-regulation of ZN428 (Figure 4C) or TGM2 (Figure 4D) worsens survival outcomes. Additionally, it has also been reported that patients with RSF1 amplification or overexpression had a significantly shorter overall survival than those without [104], although for RSF1 the p-value obtained in the Logrank Test analysis did not reveal statistical significance.

### 2.3. Effect of HMGB1 and HMGB2 Silencing on the Expression of Genes Encoding Proteins Detected in the EOC-HMGB-Interactome

**Figure 5:**
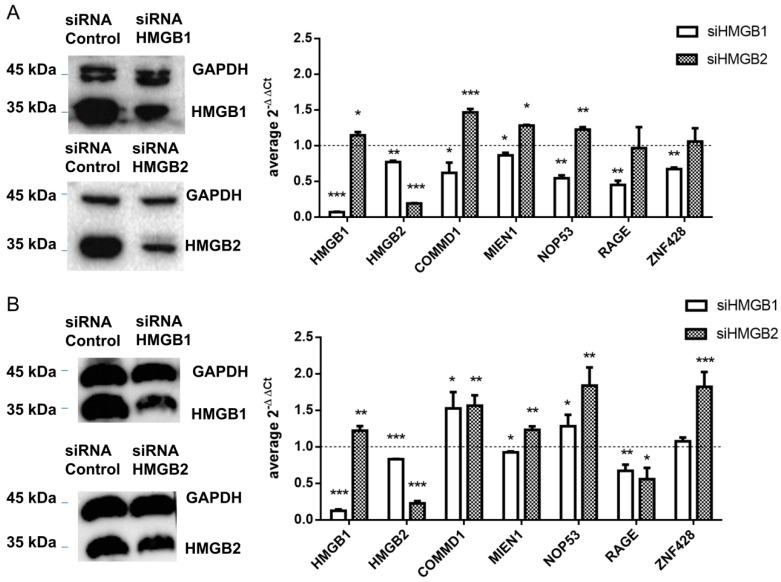
Control of gene expression by HMGB1 and HMGB2 (**A**) in SKOV-3 cells (**B**) in PEO1 cells. Left panel shows the western blot confirming HMGB1 and HMGB2 silencing; complete blots and quantification are provided in Figure S3. The right panel shows relative expression of tested genes after HMGB1 or HMGB2 silencing. The dotted line indicates no variation of relative expression in the silenced line versus the line treated with cRNA, boxes upper the line show genes over-expressed in silenced cells, and those under the line are genes under-expressed in silenced cells. * (*p* < 0.05); ** (*p* < 0.01); *** (*p* < 0.001).

HMGB1 and HMGB2 genes were silenced in SKOV-3 and PEO1 EOC cell lines and levels of mRNA from 4 detected interacting partners of HMGB1 or HMGB2 in ovary cancer, COMMD1, MIEN1, NOP53 and ZNF428, were analyzed by qRT-PCR as explained in Materials and Methods. Changes in gene expression (siHMGB/HMGB) are shown in Figure 5. In this analysis we also included RAGE, one of the membrane receptors in the extracellular signaling function of HMGB1 [97] as a positive control. HMGB1-induced signaling can activate NFκB, which can subsequently induce the expression of HMGB1 receptors [105]. Accordingly, silencing of HMGB1 causes a decrease of RAGE expression, as well as for the other tested genes in SKOV-3 (Figure 5A); in PEO1 cells this decrease was not so evident, which might be explained by a lesser degree of the silencing achieved in this cell line (Figure 5B and Figure S2). Oppositely, we observed that expression of three of the tested genes, COMMD1, MIEN1 and NOP53, was increased after HMGB2 silencing in the two EOC cell lines analyzed, while the increase in expression of ZNF428 was statistically significant only in PEO1 cells.

### 2.4. The Involvement of Proteins Detected in the EOC-HMGB-Interactome in the Response to Drugs Used in Cancer Chemotherapy

Considering that HMGB1 and HMGB2 proteins have been associated to drug resistance during cancer treatment [13,16] we also reviewed available literature to see whether the proteins detected in our interactome study could also be related to this unfavorable event in ovary cancer treatment. We found that at least 13 of these proteins had been previously cited in reference to drug resistance or sensitivity. MIEN1 has been associated to cisplatin resistance [36]. BCCIP (aliases CDKN1A or p21) is involved in resistance to carboplatin [106] and paclitaxel [107]. RSF1 is also involved in resistance to carboplatin and paclitaxel [46,49]. PCBP1 binds and stabilizes p27 mRNA and promotes cell apoptosis under paclitaxel treatment [108]. TGM2 modulates chemosensitivity of breast cancer to docetaxel [109]. DAG1 improves sensitivity to dasatinib, a tyrosine kinase inhibitor of the Src-family kinases, in EOC [45]. Besides, comparing gene expression in SKOV-3 cells and a paclitaxel resistant derived cell line (available in the GEO accession GSE54772) [110], six of the genes encoding proteins detected in our EOC-HMGB-interactomes (ATF7IP, DAG1, PCBP1, TGM2, U2AF1, and ZNF668) are expressed at higher levels in sensitive than in resistant cells, and one (WDR60) is less expressed in sensitive than in resistant cells (Figure S2).

Although the role of HMGB proteins in cisplatin resistance is widely accepted [3], there is scarce information about the role of HMGB proteins in the resistance towards its derivatives like carboplatin, or other drugs used in ovarian cancer treatment. Indeed, no data are available about the role of HMGB1 or HMGB2 in the resistance to olaparib or bevacizumab in the treatment of ovarian cancer. About the involvement of MIEN1 and NOP53 in drug resistance, only MIEN1 has been previously related to cisplatin resistance [36]. With these precedents we decided to investigate the role of HMGB1, HMGB2, MIEN1 and NOP53 in cell viability as well as in response and sensitivity to drugs currently used in ovarian cancer therapy.

We tested the effect of four compounds, used in ovary-cancer therapy in the expression of the genes HMGB1, HMGB2, MIEN1 and NOP53 in cultured cancerous SKOV-3 cells and in non-cancerous IOSE-80 ovarian cells. Each selected compound has a different mechanism of action. Carboplatin (Paraplatin^®^), a derivative of cisplatin, generates lesions in DNA, thereby inhibiting replication and transcription and leading to cell death [111,112]. Olaparib (AZD-2281, Lynparza^®^) inhibits poly ADP ribose polymerase (PARP), an enzyme necessary in DNA repair, leading to apoptosis of cancer cells [113]. Olaparib is FDA approved, for women with advanced ovarian cancer and a BRCA1/2 mutation, after they have completed the first line of platinum-based chemotherapy [114]. Bevacizumab (Avastin^®^), a humanized anti-vascular endothelial growth factor (VEGF) monoclonal antibody for cancer therapy is used as anti-angiogenic [115]; and paclitaxel (Taxol^®^), a cyclodecane first isolated from *Taxus brevifolia*, stabilizes microtubules in their polymerized form, leading to cell death [116]. Cells were exposed to drug concentrations selected according to previous studies, as explained in Materials and Methods, for 48 h. A comparative analysis between the effects caused by these drugs on SKOV-3 and non-cancerous ovarian IOS-80 cells is shown in Table 4.

Relative RNA expression after these treatments was measured by qRT-PCR in reference to cells cultured in absence of the drugs, but treated with the corresponding buffer used in the preparation of drug-solutions. In general, significant effects were more frequently observed in cancerous than in non-cancerous cells. For carboplatin or paclitaxel treatments, which are generally used in first line therapy of EOC, results indicate that they cause down regulation of the genes that are over-expressed in EOC cells (HMGB1, HMGB2 and MIEN1). Combined treatment with paclitaxel and carboplatin potentiates down-regulation of these genes in comparison to individual treatments, as deduced from the fold-changes observed. NOP53, which is expressed at lower levels in EOC than in non-cancerous cells, was up-regulated after 48 h treatment with paclitaxel. Among the genes assayed, the treatment with olaparib in cancerous cells only affected NOP53, increasing its expression (Table 4). Bevacizumab had also minor effects on HMGB2 and NOP53 expression, in this case diminishing their expression (Table 4).

### 2.5. Effect HMGB1, HMGB2, MIEN1 and NOP53 Silencing on Drug Sensitivity

**Figure 6:**
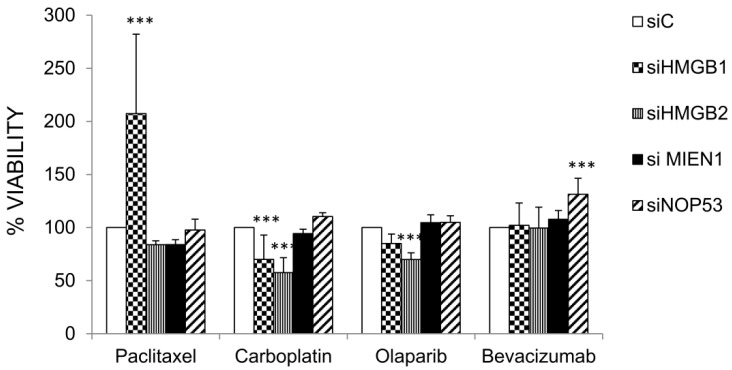
Changes in cell viability of drug-treated versus untreated SKOV-3 cells after HMGB1, HMGB2, NOP53 and MIEN1 silencing. Data about silencing of HMGB1 and HMGB2 are shown in Figure S3; and those of MIEN1 and NOP53 in Figure S4. *** (*p* < 0.001).

The genes HMGB1, HMGB2, MIEN1 and NOP53 were silenced by siRNA as described in Materials and Methods and the effect on cell viability after treatments with paclitaxel, carboplatin, olaparib and bevacizumab were compared in cells transfected with the corresponding specific siRNAs and siC (unrelated Control). Results are shown in Figure 6. Silencing of HMGB1 or HMGB2 diminished SKOV-3 cell viability after treatment with carboplatin. However, silencing of HMGB1 increased cell viability after treatment with paclitaxel. Cell viability after treatment with olaparib diminished with HMGB2 silencing. Finally, silencing of NOP53 increased cell viability after treatment with bevacizumab.

## 3. Discussion

Considering the relevance of HMGB proteins in Epithelial Ovary Cancer (EOC), we have determined for the first time the interactome of HMGB1 and HMGB2 related to this gynecological cancer. To this purpose, we have screened Y2H libraries, prepared from SKOV-3 cells and from tumor tissue diagnosed as primary transitional cell carcinoma (TCC) of the ovary, with HMGB1 and HMGB2 baits.

Supporting the functional significance of proteins detected in this EOC-HMGB-interactome study, we have found in the literature that all of them are experimentally associated to cancer hallmarks (Figure 2) and, in a high proportion, they had been directly related to ovary cancer (Figure 2). Furthermore, ROCK1 [51], PCBP1 [52], AKIP1 [53], TGM2 [54], BCCIP [50], and MIEN1 [55] proteins have been cited in relation to the epithelial to mesenchymal transition (EMT). Since EMT is an important step in carcinogenesis, which precedes metastasis, the relevance of these proteins for early EOC diagnosis is open to further analysis. Also reinforcing the significance of the interactions detected in our study in relation to clinics, data of gene expression according to Pan-Cancer Analysis of Whole Genomes (PCAWG), and corresponding to 110 tumors of ovarian adenocarcinomas, show that HMGB1, HMGB2 and >90% of their preys detected in this EOC-HMGB-interactome are up-regulated in the comparison between tumor tissue and adjacent non-tumor tissue (Table 3). Besides, according to the “Ovarian Serous Cystadenocarcinoma (TCGA)” study and using the tools from cBioportal, up-regulation of MIEN1, TGM2 or ZN428 in samples from these patients is correlated to poorer survival outcomes (Figure 4).

Although the proteins identified in the study, as well as HMGB1 and HMGB2, had been previously and independently related to EOC, the implication of a direct interaction with HMGB proteins, as part of their mechanism of action in cancer progression, had not been previously envisaged. Remarkably, this association is functionally reinforced by their confluence in a common signaling pathway; the function of HMGB1 in EOC has been previously associated to NF-kB signaling [4,5] and the functions of several of the EOC-HMGB-interactome components found in our analysis are also related to this signaling pathway. AKIP1 is a binding partner of NF-kappa B p65 subunit, which enhances the NF-kappa B-mediated gene expression [117]. MALAT1 and NF-κB signaling crosstalk during cancer and other diseases [118]. BCCIP binds to the protein LYRIC/AEG-1, which promotes tumor cell migration and invasion through activation of NF-kappaB [119]. COMMD1 inhibits NF-κB by promoting the ubiquitination and subsequent proteasomal degradation of RELA, component of NF-κB dimer, RELA/p50, bound to chromatin [120]. MIEN1 (C35) functionally enhances migration and invasion via NF-κB/Akt activity [121]. Rho-kinase isoform ROCK1 and its downstream target p38 MAPK regulate nuclear translocation of NF-κB RelA/p65 and subsequent DNA binding activity [122]. Over expression of C1qA up-regulates nuclear factor-κB reporters [123]. RSF1-overexpressing paclitaxel-resistant ovarian cancer cell lines were found to express elevated levels of genes regulated by NF-κB [46].

The interaction of HMGB2 with MIEN1 and NOP53 has been validated by co-immunoprecipitation in our study. The structure of MIEN1 and their emerging functions in relation to cancer have been recently reviewed [37], although the role of MIEN1 in pathophysiology of ovarian cancer had not been previously explored in depth. NOP53 regulates the activation of the tumor suppressor p53 when ribosome biogenesis is perturbed or DNA damage is produced [124,125,126]. Previous studies on the role of NOP53 in ovarian cancer were scarce, but down-regulation was observed in invasive serous ovarian tumors compared with benign and normal tissues [35] a feature also confirmed by our experiments in SKOV-3 cells versus noncancerous cells (Figure 3). NOP53 had been identified as a tumor suppressor downregulated in brain tumor cells [127,128]; however, in other cancers (esophagus or colon), NOP53 behaves as an oncogene that increments its expression in malignant cells [129]. Our data show that MIEN1 and NOP53 genes are regulated by HMGB1 and HMGB2 expression in EOC cell lines (Figure 5). HMGB2 negatively regulates and HMGB1 mostly positively regulates these genes, and in general the partners selected, which also correlates with the positive effect caused by HMGB2 silencing on HMGB1 expression. This could indicate that imbalance between HMGB1/HMGB2 is an important issue to consider in EOC, although further evidences are needed to clarify this mechanism.

Interestingly, we have shown in our study that HMGB1, HMGB2 and their EOC-HMGB-interactome partners MIEN1 and NOP53 are involved in the response to carboplatin, or drugs nowadays used in ovarian cancer treatment, such as bevacizumab, olaparib and paclitaxel. Expression levels of the pro-oncogenic genes HMGB1, HMGB2, and MIEN1 are down-regulated after treatment with paclitaxel, carboplatin or a combination of both (Table 4). Accordingly, HMGB1 or HMGB2 silencing decrease cell-viability of SKOV-3 cells exposed to carboplatin (Figure 6). Our data support the role of HMGB1 in resistance to carboplatin that was previously reported [15]. No data were previously available about the role of HMGB1 or HMGB2 in the resistance to paclitaxel, olaparib or bevacizumab in EOC treatment, although HMGB1 had been reported as a possible prognosis biomarker of bevacizumab treatment in non-small-cell lung cancer [130] and bevacizumab and HMGB1 have been related to malignant mesothelioma [131]. We have not detected changes in HMGB1 gene expression or effects of HMGB1 silencing in ovarian cancerous cells viability after bevacizumab treatment in the assayed conditions. However, we have found that HMGB1 is related to sensitivity to paclitaxel and HMGB2 is related to sensitivity to olaparib. NOP53, considered a tumor suppressor in brain tumor cells [127,128], is overexpressed after treatment with paclitaxel and bevacizumab, and NOP53 silencing decrease sensitivity to bevacizumab in SKOV-3 cells. According to our data, downregulation of HMGB1 causes loss of response to paclitaxel; down regulation of HMGB1 or HMGB2 increases sensitivity to carboplatin and, only in the case of HMGB2, slightly increases sensitivity to olaparib; finally, down regulation of NOP53 causes a moderate loss of response to bevacizumab. Levels of these genes might be analyzed in the clinical laboratory to guide treatment strategy in order to improve prognosis and avoid resistances.

## 4. Materials and Methods

### 4.1. Yeast Two Hybrid Methodology

HMGB1 and HMGB2 interacting partners were identified using Matchmaker Gold Yeast Two-Hybrid System (Clontech. Fremont, CA, USA). *Sacchacomyces cerevisiae* strains were Y187 (MATα, ura3-52, his3-200, ade2-101, trp1-901, leu2-3, 112, gal4Δ, gal80Δ, met-, URA3::GALuas-GAL1TATA-LacZ MEL1) and Y2HGold (MATa, trp1-901, leu2-3, 112, ura3-52, his3-200, gal4Δ, gal80Δ, LYS2::GAL1UAS-GAl1TATA-HIS3, GAL2UAS-GAL2TATA-ADE2, URA3::MEL1UAS-MEL1TATA-AUR1-C-MEL1). This is a very reliable Y2H system in which the three used promoters, controlling the four reporter genes HIS3, ADE2, AUR1-C and MEL1 in Y2HGold, are unrelated except for the short sequence in the upstream activation site (UAS) that are specifically bound by the Gal4 DNA binding domain. Thus, library proteins that interact with unrelated sequences, flanking or within the UAS, (i.e., false positives) are automatically screened out.

RNAs from human samples used to prepare the Y2H libraries were provided by Biobanco de Andalucía (Granada, Spain). Tumor and paired non-tumor tissue were obtained from a 63-year-old woman diagnosed with grade III ovarian transitional cell carcinoma without previous chemotherapy treatment. RNA was extracted from frozen tissue sections in OCT (Optimal Cutting Temperature) compound, using the Qiacube robot (Qiagen. Hilden, Germany) based on ion exchange columns with silica membrane. RNA was obtained with the miRNeasy mini kit (Qiagen, Hilden, Germany). The samples were finally treated with RNase-Free DNAase (Qiagen, Hilden, Germany). The quantity of RNA obtained was evaluated at 260 nm and 280 nm by spectrophotometry using the Infinite F200 equipment (Tecan Group Ltd. Männedorf. Switzerland) with a Nanoquant plate; finally, the integrity of the samples was evaluated by the 2200 Tape Station apparatus (Agilent Technologies, Inc. Santa Clara, CA, USA), being the RIN (RNA Integrity Number) parameter greater than 8. Total RNA from the ovarian cell line SKOV-3, was also used to prepare cDNA libraries. Library construction, bait construction and Yeast Two-Hybrid library screening were done as recommended by the vendor of the Matchmaker Gold Yeast Two-Hybrid System (Clontech, Mountain View, CA, USA) and details are already published [132]. After plasmid rescue, inserts were sequenced with primer T7 (5′-TAATACGACTCACTATAGGG-3′). Homology searches were done with BlastN and BlastX at NCBI (https://blast.ncbi.nlm.nih.gov/ accessed on 02-02-2020).

### 4.2. Cell Lines, Treatments and Cell Viability Assays

The SKOV-3 and PEO1 cell lines (originally derived from human EOC) and regularly tested for mycoplasma by Eurofins Scientific (Luxembourg, Luxembourg), were grown in McCoy’s-5A or RPMI-1640 medium, respectively, supplemented with 10% heat-inactivated fetal bovine serum and 1% penicillin-streptomycin (Thermo Fisher Scientific Inc. Waltham, MA, USA). The non-cancerous immortalized human ovarian cell line IOS3-80 (RRID:CVCL_5546) was obtained from Canadian Ovarian Tissue Bank (University of British Columbia, Vancouver, BC, Canada) and grown in RMPI-1640 medium supplemented as described above. Cells were cultured in a humidified incubator at 37 °C at 5% CO_2_.

SKOV-3 and IOSE-80 cells at 80% confluence were exposed during 48 h to different treatments, using drug concentrations and conditions selected according to previous studies. Paclitaxel was used at 25 μM [133]; carboplatin at 25 μg/mL [15]; olaparib to 2 μM [134]; and bevacizumab at 100 µg/mL [135] Paclitaxel was purchased to Sigma Aldrich Inc. (St. Louis, MO, USA) and olaparib, bevacizumab and carboplatin were provided by the Pharmacy Service of the Teresa Herrera hospital (INIBIC). In parallel, cells were grown with the same amount of vehicle-buffer used to prepare drug solutions, or with an unspecific IgG not directed to vascular endothelial growth factor as control of bevacizumab treatment. Cell viability-cytotoxicity assays were done using the Cell Counting Kit-8, CCK-8 (Tebu-Bio. Le-Perray-en-Yvelines, France).

### 4.3. Cross-Linking and HMGB2 Co-Immunoprecipitation

After reaching 70–80% confluence of SKOV-3 and PEO1 cultures, medium was removed and substituted by medium without fetal bovine serum and supplemented with 1% formaldehyde used as cross-linker. The cells were incubated with formaldehyde during 10 min at room temperature. To stop the cross-linking reaction, the medium with formaldehyde was substituted with a solution containing 0.125 M glycine in PBS (Phosphate Buffered saline) pH 7.4 (NZYTech, Lda. Lisbon, Portugal) and incubated during 5 min at room temperature. Cells were washed three times with PBS and harvested by scraping. After subsequent collection by centrifugation at 1200 rpm for 10 min at 4 °C, cells were re-suspended in lysis buffer (50 mM Tris-HCl pH 8, 150 mM NaCl, 0.1% NP-40, 1 mM EDTA, 2 Mm MgCl_2_) containing a cocktail of EDTA-free protease inhibitors (Roche Diagnostics, Laval, QC, Canada). Cell lysates were clarified for 15 min at 14000 rpm to pellet cell debris. Supernatants were collected and protein quantified using the Bradford reagent. 16 mg of total protein extracts from cells were immunoprecipitated (IPs) using 10 µg HMGB2 rabbit polyclonal ab67282 (Abcam, Cambridge, UK) bound to 50 μL dynabeads-protein A (Thermo Fisher Scientific Inc. Waltham, MA, USA) following manufacturer’s instructions. The presence of MIEN1 and NOP53 in the immunoprecipitations (IPs) was confirmed by western blot using the antibodies against MIEN1 (PA1-31180 from Thermo-Fisher Scientific Inc. Waltham, MA, USA) and NOP53, (sc517088 Santa Cruz, Dallas, TX, USA). After second incubation with 1:5000 G-protein HRP-linked (18-161.Millipore-Merck-KGaA, Darmstadt, Germany), 5% (w/v) non-fat milk diluted in PBST, PSB containing 0.1% Tween 20 (P1379 from Sigma Aldrich Inc.) was used as blocking solution. Western blots were developed using LuminataTMCrescendo Western HRP Substrate (Millipore Corporation. Burlington, MA, USA), and visualized in a ChemiDocTM imager (Bio-Rad Laboratories. Hercules, CA, USA).

### 4.4. Gene Expression Analysis by Quantitative Retrotranscription and Polymerase Chain Reaction (qRT-PCR)

RNA samples from cell cultures were obtained using GeneJET RNA Purification Kit (Thermo Fisher Scientific Inc., Waltham, MA, USA). The samples were treated with RNase-Free DNAase (Thermo Fisher Scientific Inc., Waltham, MA, USA) and purified using GeneJET RNA Cleanup and Concentration kit (Thermo Fisher Scientific Inc. Waltham, MA, USA). RNA from human ovarian surface epithelial cells (HOSEpiC) was provided by Innoprot (Derio, Vizcaya, Spain). RNA samples were retro-transcribed into cDNA and labeled with the KAPA SYBR FAST universal one-step qRT-PCR kit (Kappa Biosystems Inc., Woburn, MA, USA). The primers for qPCR were designed with the characteristics shown in Table S1 (Supplementary Material). Reaction conditions for thermal cycling were: 42 °C for 5 min, 95 °C for 5 s, 40 cycles of 95 °C for 3 s and finally 60 °C for 20 s. The ECO Real-Time PCR System was used for the experiments (Illumina Inc., San Diego, CA, USA), and relative expression was calculated by the 2^−ΔΔCt^ method [136]. A *t*-test was used to check the statistically significance of differences between samples (*p* < 0.05 at least). The relative expression of selected genes were calculated by referring to the mRNA levels of the housekeeping gene, GADPH, which had been verified as being expressed constitutively under the assay conditions. For valid quantification using the 2^−ΔΔCt^ method, the target and reference primer pairs were previously tested for PCR efficiencies and differed by <10%. At least, three independent biological replicas and two technical replicas were made for each.

### 4.5. siRNA Silencing

siRNAs directed against each mRNA and unspecific controls were purchased. siRNA-HMGB1 (s20254 Silencer Select) and siRNA-HMGB2 (s6650) from Life technologies (Thermo Fisher Scientific Inc., Waltham, MA, USA); siRNA-MIEN1 (S228354), siRNA-NOP53, (S26871) and siRNAControl2 (4390846) from Ambion Inc. (Thermo Fisher Scientific Inc., Waltham, MA, USA). Transfection of cells with siRNAs was done using Lipofectamine^®^ 2000 (Life-Technologies-Invitrogen. Thermo Fisher Scientific Inc., Waltham, MA, USA) and following the protocol recommended by the vendor. Silencing was verified by qRT-PCR, with the methods described in the previous section, and Western blot using the antibodies against HMGB1 and HMGB2, NOP53 and MIEN1 already described and anti-GAPDH (60004-I-Ig from Proteintech. Manchester, UK) used for loading control. After second incubation with 1:5000 G-protein HRP-linked (18–161, Millipore-Merck-KGaA), western blot was developed as above described.

### 4.6. Survival Analysis

The Overall Survival Kaplan-Meier Estimate analysis was performed through cBioPortal (http://www.cbioportal.org/ accessed on 3-6-2020) using the databases Ovarian Serous Cystadenocarcinoma (TCGA, Provisional), composed of 606 samples. Results obtained for the genes giving Logrank Test *p* < 0.05 were selected for discussion.

## 5. Conclusions

In conclusion, results from our EOC-HMGB interactome study provides a set of proteins highly correlated with cancer hallmarks, EMT, ovarian cancer, NF-kB signaling and, the expression of some of them has been previously associated to patient’s survival. We have experimentally probed that HMGB1, HMGB2 and two of their partners, MIEN1 and NOP53 are also involved in the response of ovarian cancer cells to several drugs used in chemotherapy against EOC. Although clinical studies are needed before translation to early diagnosis and prognosis of patients, these proteins found in our EOC-HMGB-Interactome study are in the focus for the search of biomarkers and therapeutic targets in the fight against EOC disease.

## Figures and Tables

**Table 1:** Clones from the SKOV-3 libraries, which interact with HMGB1 or HMGB2 in the Y2H assays.

Interacting Partner	Bait	Aminoacids	Uniprot CODE	Brief Functional Description According to Uniprot (http://www.uniprot.org/uniprot) (accessed on 05-05-2020)
AKIP1	HMGB1	29–210	Q9NQ31	A-kinase-interacting protein 1 that regulates the effect of the cAMP-dependent protein kinase signaling pathway on the NF-kappa-B activation cascade.
KRT7	HMGB1	102–289	P08729	Keratin, type II cytoskeletal 7 that blocks interferon-dependent interphase and stimulates DNA synthesis in cells.
MALAT1	HMGB1	lncRNA		
ATF7IP	HMGB1	8–250	Q6VMQ6	Recruiter that couples transcriptional factors to general transcription apparatus and thereby modulates transcription regulation and chromatin formation. Facilitates telomerase TERT and TERC gene expression by SP1 in cancer cells
UHRF2	HMGB1	157–277	Q96PU4	E3 ubiquitin-protein ligase UHRF2 that is an intermolecular hub protein in the cell cycle network. Through cooperative DNA and histone binding, may contribute to a tighter epigenetic control of gene expression in differentiated cells.
WDR60	HMGB1	170–336	Q8WVS4	WD repeat-containing protein 60.
BCCIP	HMGB2	8–257	Q9P287	BRCA2 and CDKN1A-interacting protein that is required for microtubule organizing and anchoring activities during interphase.
COMMD1	HMGB2	2–189	Q8N668	COMM domain-containing protein 1. Proposed scaffold protein that is implicated in diverse physiological processes and whose function may be in part linked to its ability to regulate ubiquitination of specific cellular proteins.
NOP53 (alias GLTSCR2 or PICT1)	HMGB2	186–453	Q9NZM5	Ribosome biogenesis protein NOP53. Originally identified as a tumor suppressor, it may also play a role in cell proliferation and apoptosis by positively regulating the stability of PTEN, thereby antagonizing the PI3K-AKT/PKB signaling pathway.
MIEN1 (alias C35)	HMGB2	1–116	Q9BRT3	Migration and invasion enhancer 1 that increases cell migration by inducing filopodia formation at the leading edge of migrating cells. Plays a role in regulation of apoptosis, possibly through control of CASP3.
ROCK1	HMGB2	141–197	Q13464	Rho-associated protein kinase 1 that is a key regulator of actin cytoskeleton and cell polarity.
U2AF1	HMGB2	35–202	Q01081	Splicing factor U2AF 35 kDa subunit, that plays a critical role in both constitutive and enhancer-dependent splicing by mediating protein-protein, and protein-RNA interactions required for accurate 3′-splice site selection.
ZNF668	HMGB2	16–239	Q96K58	Zinc finger protein 668

**Table 2:** Clones from the cancerous ovarian tissue libraries, which interact with HMGB1 or HMGB2 in the Y2H assays.

Interacting Partner	Bait	Aminoacids	Uniprot Code	Brief Functional Description According to Uniprot (http://www.uniprot.org/uniprot) (accessed on 05-05-2020)
C1QA	HMGB1	47–177	P02745	Complement C1q subcomponent subunit A
DAG1	HMGB1	311–516	Q14118	Dystroglycan. The dystroglycan complex is involved in a number of processes including laminin and basement membrane assembly, sarcolemmal stability, cell survival, peripheral nerve myelination, nodal structure, cell migration, and epithelial polarization
RPL29	HMGB1	36–143	P47914	60S ribosomal protein L29
RSF1	HMGB1	616–799	Q96T23	Remodeling and spacing factor 1 required for assembly of regular nucleosome arrays by the RSF chromatin-remodeling complex
TGM2	HMGB1	377–480	P21980	Transmembrane gamma-carboxyglutamic acid protein 2
COMMD1	HMGB2	4–189	Q8N668	COMM domain-containing protein 1. Proposed scaffold protein that is implicated in diverse physiological processes and whose function may be in part linked to its ability to regulate ubiquitination of specific cellular proteins.
MIEN1 (alias C35)	HMGB2	1–116	Q9BRT3	Migration and invasion enhancer 1 that increases cell migration by inducing filopodia formation at the leading edge of migrating cells. Plays a role in regulation of apoptosis, possibly through control of CASP3.
PCBP1	HMGB2	26–202	Q15365	Poly (rC)-binding protein 1. Single-stranded nucleic acid binding protein that binds preferentially to oligo dC.
TBC1D25	HMGB2	309–366	Q3MII6	TBC1 domain family member 25. Acts as a GTPase-activating protein specific for RAB33B. Involved in the regulation of autophagosome maturation.
ZFR	HMGB2	294–722	Q96KR1	Zinc finger RNA-binding protein. Involved in postimplantation and gastrulation stages of development. Involved in the nucleocytoplasmic shuttling of STAU2.
ZNF428	HMGB2	153–188	Q96B54	Zinc finger protein 428.

**Table 3:** Differential expression of HMGB1, HMGB2 and their interacting partners in healthy individuals and ovary cancer patients. mRNA levels obtaining by RNAseq are expressed in TPM (Transcripts per million).

Gene Name	Ovarian Adenocarcinoma	Normal Ovary Tissue (GTEx)	Ratio Cancerous/Healthy
AKIP1	45	28	1.6
ATF7IP	46	14	3.3
BCCIP	131	33	4.0
C1QA	613	49	12.5
COMMD1	65	17	3.8
DAG1	264	51	5.2
HMGB1	524	153	3.4
HMGB2	453	100	4.5
KRT7	1258	0,7	1797
MALAT1	244	886	0.3
MIEN1	144	23	6.3
NOP53	427	576	0.7
PCBP1	1554	386	4.0
ROCK1	42	21	2.0
RPL9	2526	1540	1.6
RSF1	37	13	2.8
TBC1D25	40	20	2.0
TGM2	118	50	2.4
U2AF1	81	42	1.9
UHRF2	35	28	1.3
WDR60	34	21	1.6
ZFR	149	57	2.6
ZNF428	197	64	3.1
ZNF668	25	5	5.0

**Table 4:** Comparative effect of treatments on gene expression in cancerous (SKOV-3) versus non-cancerous (IOSE80) ovarian cells.

			SKOV-3					IOSE80				
Treatment	Time	GENE	2^−∆∆Ct^	SD	Effect	CF	*p* Value	2^−∆∆Ct^	SD	Effect	CF	*p* Value
Taxol	48 h	HMGB1	0.23	0.05	Down	4.44	7.50 × 10^−08^	0.03	0.01	Down	30.76	4.92 × 10^−04^
Taxol	48 h	HMGB2	0.36	0.11	Down	2.80	6.11 × 10^−09^	0.77	0.10	Ns	–	9.47 × 10^−02^
Taxol	48 h	MIEN1	0.23	0.07	Down	4.40	1.14 × 10^−10^	0.005	0.003	Down	215.5	1.18 × 10^−03^
Taxol	48 h	NOP53	8.29	3.03	Up	8.29	2.62 × 10^−09^	3.01	0.29	Up	3.01	6.60 × 10^−04^
Carboplatin	48 h	HMGB1	0.17	0.08	Down	5.90	2.31 × 10^−03^	1.74	0.76	Ns	–	3.48 × 10^−01^
Carboplatin	48 h	HMGB2	0.24	0.02	Down	4.12	3.07 × 10^−05^	1.26	0.22	Ns	–	2.31 × 10^−01^
Carboplatin	48 h	MIEN1	0.11	0.03	Down	9.49	6.78 × 10^−05^	0.74	0.15	Ns	–	3.04 × 10^−01^
Carboplatin	48 h	NOP53	0.49	0.08	Down	2.06	4.09 × 10^−03^	1.11	0.34	Ns	–	6.94 × 10^−01^
Olaparib	48 h	HMGB1	0.66	0.16	Ns	–	1.24 × 10^−02^	0.83	0.12	Ns	–	1.65 × 10^−01^
Olaparib	48 h	HMGB2	1.12	0.35	Ns	–	4.90 × 10^−01^	0.72	0.09	Ns	–	2.82 × 10^−02^
Olaparib	48 h	MIEN1	1.72	0.33	Ns	–	9.61 × 10^−01^	0.66	0.20	Ns	–	9.92 × 10^−02^
Olaparib	48 h	NOP53	12.27	2.97	Up	12.27	2.41 × 10^−06^	0.68	0.08	Ns	–	7.38 × 10^−02^
Bevacizumab	48 h	HMGB1	0.68	0.11	Ns	–	4.19 × 10^−02^	0.17	0.11	Ns	–	2.72 × 10^−02^
Bevacizumab	48 h	HMGB2	0.56	0.09	Down	1.78	3.15 × 10^−04^	0.49	0.14	Down	2.05	8.52 × 10^−03^
Bevacizumab	48 h	MIEN1	0.89	0.13	Ns	–	2.37 × 10^−01^	0.06	0.05	Ns	–	1.62 × 10^−02^
Bevacizumab	48 h	NOP53	0.31	0.07	Down	3.22	6.75 × 10^−08^	0.98	0.49	Ns	–	7.41 × 10^−01^
Taxol + Carboplatin	48 h	HMGB1	0.05	0.02	Down	19.05	4.55 × 10^−04^	Nt	Nt	Nt	Nt	Nt
Taxol + Carboplatin	48 h	HMGB2	0.10	0.05	Down	10.37	1.02 × 10^−03^	Nt	Nt	Nt	Nt	Nt
Taxol + Carboplatin	48 h	MIEN1	0.04	0.01	Down	25.66	8.60 × 10^−05^	Nt	Nt	Nt	Nt	Nt
Taxol + Carboplatin	48 h	NOP53	1.63	0.50	Ns	–	3.76 × 10^−01^	Nt	Nt	Nt	Nt	Nt

Down: the treatment causes diminished mRNA expression, Up: the treatment causes increased mRNA expression. Ns: the effect is not significant having a *p* value > 0.01. Nt: not tested. CF: Change fold (treated *versus* non-treated cells); 2^−∆∆Ct^ Relative quantification that relates the PCR signal of the target transcript in a treatment group to that of an untreated control.

## Supplementary Materials

The following are available online at https://www.mdpi.com/2072-6694/12/9/2435/s1, Table S1: Oligonucleotides used in this study, Figure S1: Co-immunoprecipitation of HMGB2 and MIEN1 or NOP53, Figure S2: Differential expression of components of the EOC-HMGB-Interactome in normal *versus* paclitaxel resistant cells, Figure S3: Quantification of HMGB1 and HMGB2 silencing, Figure S4: Quantification of MIEN1 and NOP53 silencing in SKOV-3 cells.
