# Supplementary material for: The HMGB1-2 Ovarian Cancer Interactome. The Role of HMGB Proteins and Their Interacting Partners MIEN1 and NOP53 in Ovary Cancer and Drug-Response
Source: Cancers (Basel). 2020 Aug 27;12(9):2435. doi: 10.3390/cancers12092435 (PMC7564582; doi:10.3390/cancers12092435)
Supplement: Supplementary file 1 [file cancers-12-02435-s001.pdf]

cancers

Table S1. Oligonucleotides used in this study.

Gene Name	Sequence	Tm (°C)	Hybridization Site	Amplicon Size (bp)
COMMD1	F: 5´-CAGCTATATCCAGAGGTGCCAC-3´	62.1	112–133	109
COMMD1	R: 5´-CCTCCAGCTGGTTGAAATCC-3´	59.4	233–253	
GAPDH	F:5′-CCTCCTGCACCACCAACTG-3′	61.2	449–467	102
GAPDH	R:5′-TGGCAGTGATGGCATGGA-3′	59.5	533–550	
HMGB1	F:5′-TCAAAGGAGAACATCCTGGCC-3′	60.6	338–358	87
HMGB1	R:5′- GCTTGTCATCTGCAGCAGTGTT-3′	62.5	403–424	
HMGB2	F:5′-GAGCAGTCAGCCAAAGATAAACAA-3′	60.4	403–426	111
HMGB2	R:5′-TCCTGCTTCACTTTTGCCCTT-3′	61.0	493–513	
KRT7	F:5′-TGAATGATGAGATCAACTTCCTCAG-3′	59.2	653–677	75
KRT7	R:5′-TGTCGGAGATCTGGGACTGC-3′	61.9	708–727	
MIEN1	F:5′-TTGGGGGCAGGAGAGAGAC-3′	61.2	519–537	107
MIEN1	R:5′-TTACCGAGGCGAAGAGTGG-3′	59.7	607–625	
NOP53	F: 5′-ACCAGTTCCTGGAAGACGTG-3′	60.2	182–201	109
NOP53	R: 5′-CCTTTTTCCTTGGAGCCAG-3′	56.7	272–290	
RAGE	F:5′-TGTGTGGCCACCCATTCC-3′	60.5	901–918	109
RAGE	R:5′-CTGATCCTCCCACAGAGCC-3′	59.7	991–1009	
ZNF428	F: 5´-´CCCGAGCATTCCTCTGATTC-3	58.7	79–98	95
ZNF428	R: 5´-TCGTCAGTGGTCTCCTCTTC-3´	59.0	154–173	

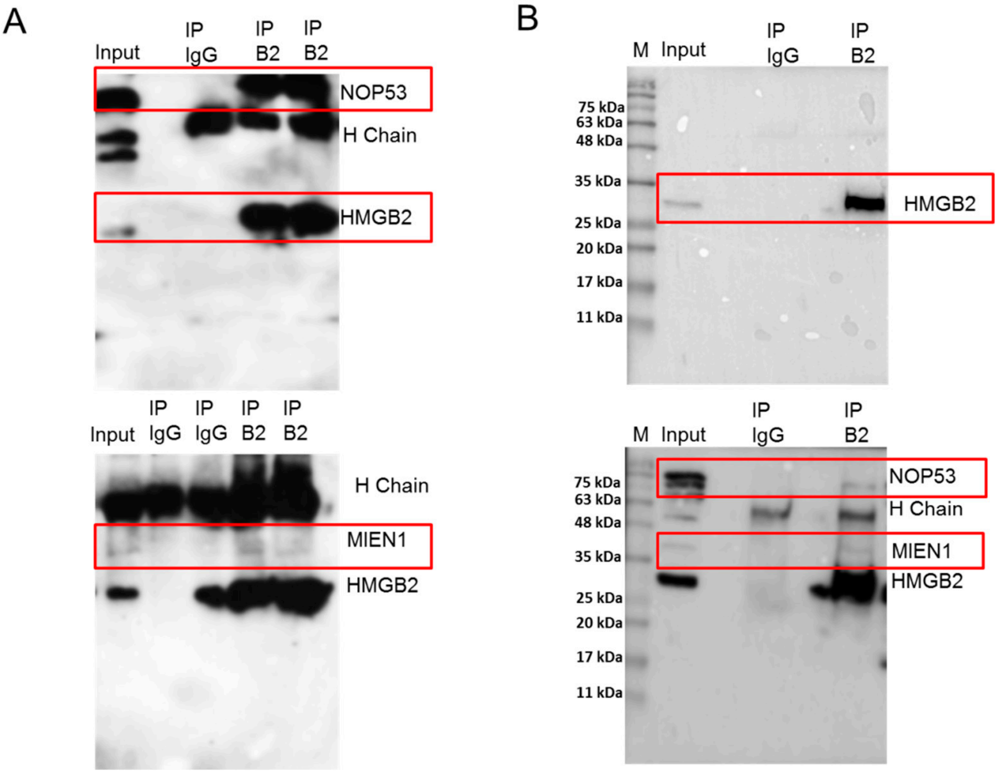

Figure S1. Co-immunoprecipitation. Complete Western blots of images shown in Figure 1. (**A**) SKOV-3 cells; upper part with HMGB2 and NOP53 antibodies, and lower part incubated with HMGB2 and MIEN1 antibodies. (**B**) PEO1 cells; upper part only incubated with HMGB2 antibody, and lower part incubated with the three antibodies.

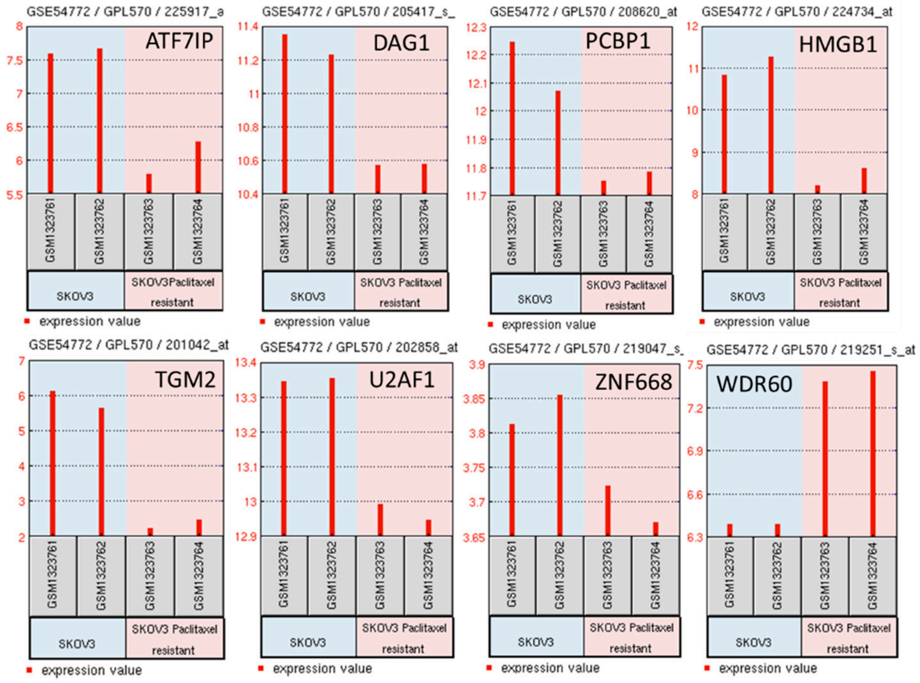

Figure S2. Differential expression of components of the EOC-HMGB-Interactome in normal versus paclitaxel resistant cells. SKOV-3 (blue shadow) versus derived SKOV-3 paclitaxel resistant cells (pink shadow) according to public data in GEO GSE54772 Expression value: TPM units.

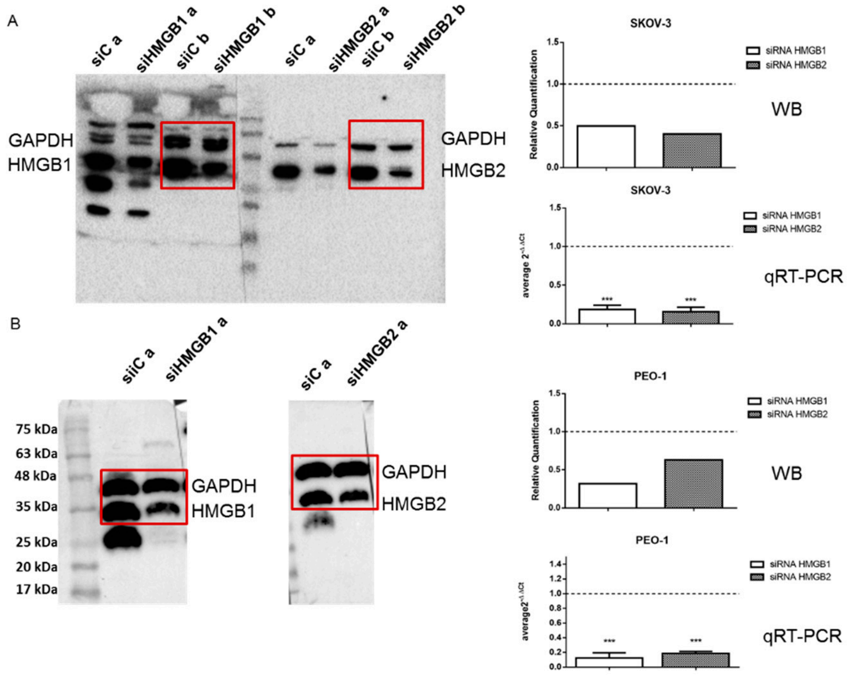

Figure S3. Quantification of HMGB1 and HMGB2 silencing. Complete blots of images shown in Figure 3. (**A**) SKOV-3 cells. (**B**) PEO1 cells. Relative quantification in cells treated with the specific siRNA or siControl (siC) is shown on the left panels as calculated from Western blots (WB) or measuring relative mRNA levels (qRT-PCR). a, samples extracted after 48 h of silencing; b, after 96 h. Bands shown in figure 5 are inside red boxes.

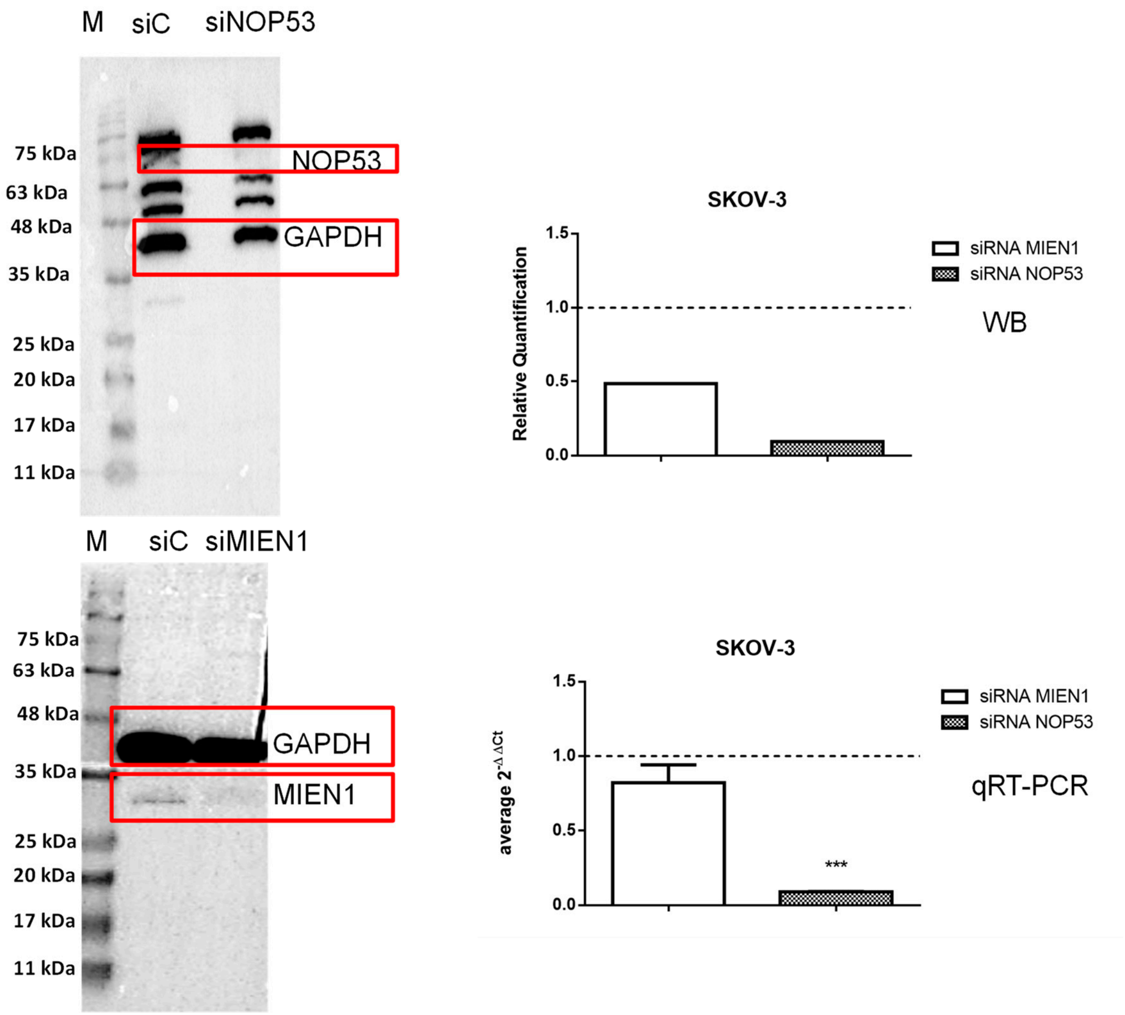

Figure S4. Quantification of MIEN1 and NOP53 silencing in SKOV-3 cells. Relative quantification in cells treated with the specific siRNA or siControl (siC) is shown on the left panels as calculated from Western blots (WB) or measuring relative mRNA levels (qRT-PCR).
